# Liraglutide improves lipid metabolism by enhancing cholesterol efflux associated with ABCA1 and ERK1/2 pathway

**DOI:** 10.1186/s12933-019-0954-6

**Published:** 2019-11-09

**Authors:** Ya-Ru Wu, Xiao-Yun Shi, Chun-Yan Ma, Yue Zhang, Rui-Xia Xu, Jian-Jun Li

**Affiliations:** 1 Division of Dyslipidemia, State Key Laboratory of Cardiovascular Disease, National Center for Cardiovascular Disease, Fu Wai Hospital, Chinese Academy of Medical Sciences and Peking Union Medical College, Beijing, 100037 China; 2 Division of Endocrinology, Beijing Chaoyang Integrative Medicine Emergency Medical Center, Beijing, 100022 China

**Keywords:** Liraglutide, db/db mice, High-fat diet, Reverse cholesterol transport, High glucose, ABCA1, Molecular mechanism

## Abstract

**Background:**

Reverse cholesterol transport (RCT) is an important cardioprotective mechanism and the decrease in cholesterol efflux can result in the dyslipidemia. Although liraglutide, a glucagon like peptide-1 analogue, has mainly impacted blood glucose, recent data has also suggested a beneficial effect on blood lipid. However, the exact mechanism by which liraglutide modulates lipid metabolism, especially its effect on RCT, remain undetermined. Hence, the aim of the present study was to investigate the potential impacts and potential underlying mechanisms of liraglutide on the cholesterol efflux in both db/db mice and HepG2 cells.

**Methods:**

Six-week old db/db mice with high fat diet (HFD) and wild type mice were administered either liraglutide (200 μg/kg) or equivoluminal saline subcutaneously, twice daily for 8 weeks and body weight was measured every week. After the 8-week treatment, the blood was collected for lipid evaluation and liver was obtained from the mice for hematoxylin–eosin (HE) staining, red O staining and Western blotting. Cholesterol efflux was assessed by measuring the radioactivity in the plasma and feces after intraperitoneal injection of ^3^H-labeled cholesterol. HepG2 Cells were treated with different concentrations of glucose (0, 5, 25, and 50 mmol/L) with or without liraglutide (1000 nmol/L) for 24 h. The intracellular cholesterol efflux was detected by BODIPY-cholesterol fluorescence labeling. Real-time PCR or Western blotting was used to examine the expression levels of ABCA1, ABCG1 and SR-B1.

**Results:**

Liraglutide significantly decreased blood glucose, serum total cholesterol (TC), triglyceride (TG) and low-density lipoprotein cholesterol (LDL-C). It also reduced liver lipid deposition in db/db mice fed with HFD. Moreover, the movement of ^3^H-cholesterol from macrophages to plasma and feces was significantly enhanced in db/db mice fed with HFD after liraglutide adminstration. In vitro study, liraglutide could promote the cholesterol efflux of HepG2 cells under high glucose, and also increase the expression of ABCA1 by activating the ERK1/2 pathway.

**Conclusions:**

Liraglutide could improve lipid metabolism and hepatic lipid accumulation in db/db mice fed with HFD by promoting reversal of cholesterol transport, which was associated with the up-regulation of ABCA1 mediated by the ERK1/2 phosphorylation.

## Background

Type 2 diabetes mellitus (DM), known as one of the commonest chronic diseases, is a type of disease in which clinical manifestations are mainly heterogeneity of insulin resistance with relative insulin deficiency. Patients with DM and coronary heart disease (CAD) is more severe than ones with CAD alone and the risk of cardiovascular events is much higher [1]. One of the reasons for this phenomenon is that hyperglycemia can cause dyslipidemia and accelerate atherosclerosis (AS) development [2]. As we well known, elevated blood glucose is a risk factor for atherosclerotic cardiovascular disease (ASCVD), which can alter a variety of cellular functions in the body and exert worse impact on the pathological process of AS [3, 4]. Besides, it has well been established that dyslipidemia is also an causal and independent risk factor of ASCVD. The interactions of abnormal glucose and lipid metabolism can exert a synergistic effect by activating a series of vascular cell pathways. For example, Hao et al. [5] found that high glucose could affect the transcription and translation of SREBP-1 through the activation of PI3K/Akt pathway, and ultimately up-regulate fatty acid synthase and acetyl-CoA hydroxylase, and increase the synthesis of fatty acids in epithelial cells and lead to lipid droplet deposition. Additionally, another similar study [6] reported that high glucose could increase lipid accumulation in mesangial cells by damaging cholesterol transporters. All of these studies strongly indicate that DM accompanied with dyslipidemia can result in more severe damage in vascular network.

Reverse cholesterol transport (RCT) refers to the process by which cholesterol from lipid-loaded peripheral cells passages through the plasma high-density lipoprotein (HDL) compartment to the liver and is excreted via the feces [7]. It has been reported that three pathways may mediate the cholesterol efflux: simple diffusion, facilitated diffusion mediated by scavenger receptor B1 (SR-BI), and efflux mediated by the ATP binding cassette transporter A1 (ABCA1) or cassette transporter G1 (ABCG1) in the presence of extracellular acceptors, such as lipid-poor apoproteins or more mature HDL, respectively [7–9]. A series of studies have confirmed that cholesterol efflux can occur in fibroblasts, adipocytes, macrophages and other peripheral tissue cells [10–12]. Functionally, RCT plays a very important role in lipid metabolism and abnormal RCT pathway reduces cholesterol efflux, accelerates lipid deposition and promotes the formation of AS. Thereby, RCT is, currently, a hot topic for the basic and clinical atherosclerosis research. Currently, promoting macrophage RCT has become the research direction of HDL anti-atherosclerosis [13].

Liraglutide, a Glucagon-like peptide-1 (GLP-1) analogue, is a novel therapeutic drug for the treatment of DM [14–16]. Compared with traditional oral hypoglycemic agents, liraglutide has a pleiotropic effect on glucolipid metabolism, which is closely associated with anti-atherosclerosis [17]. Previous studies have suggested that liraglutide exhibits the beneficial actions on islet β cells, body weight, and cardiovascular function [18–21]. A large number of studies have shown that effective control of blood glucose levels in patients with DM can significantly reduce the risk of adverse cardiovascular events [22, 23]. However, data regarding the role of liraglutide in RCT is currently limited. Therefore, this study aimed to investigate the effect of liraglutide on cholesterol efflux in db/db mice with high-fat diet (HFD) and HepG2 cells under high glucose conditions for the purpose of elucidating the potential mechanisms.

## Methods

### Materials

Male db/db mice (n = 48, 5 weeks old, BKS-Leprem2Cd479/Nju, Leprdb mut/mut) and male C57BL/6J mice (n = 12, 5 weeks old, Leprdb wt/wt) were purchased from Model Animal Research Center of Nanjing University. Liraglutide was purchased from Novo Nordisk, Bagsværd, Denmark. The human hepatoma cell line, HepG2, obtained from Cell Resource Center, IBMS, CAMS/PUMC (Beijing, China), U0126 (ERK1/2 inhibitor) was purchased from Cell Signaling Technology (Beverly, MA). Anti-ABCA1, Anti-ABCG1, Anti-SR-B1, and GAPDH antibodies were obtained from Abcam (Cambridge, UK). Antibodies against phospho-ERK1/2, total ERK1/2, were purchased from Cell Signaling Technology (Beverly, MA).

### Animal treatment

All animals were maintained in an air-conditioned environment with a controlled temperature at 22 ± 2 °C and 50–60% relative humidity under 12-h shift of the light–dark cycle. After an adaptation period of 1 week, all mice were randomly divided into the five groups: wild type + normal diet (WT + ND, n = 12), db/db + ND (n = 12), db/db + High-fat diet (HFD, n = 12), db/db + HFD + liraglutide (LIRA, n = 12), and db/db + HFD + Atorvastatin (AT) (n = 12). Mice were administered either liraglutide (200 μg/kg) or equivoluminal 0.9% saline subcutaneously, twice daily for 8 weeks. Diabetic mice fed with HFD were orally administered with atorvastatin (20 mg/kg/d) for 8 weeks as a positive control group. During this period, body weight was determined weekly and the fasting blood glucose levels were measured every 4 weeks. After 8-week treatment, mice were euthanized using 1% sodium pentobarbital (50 mg/kg) after a 4-hour fast. The eyeballs were removed and blood samples were collected. The subsequent serum was used to determine blood lipid parameters. The livers were washed using iced saline for further analysis. All experiments were approved by the Ethics Committee for Animal Care and Research at Fuwai hospital (Beijing, China).

### Lipids analysis

According to the manufacturer’s instructions, serum was prepared from each blood sample by centrifugation at 3500 rpm for 10 min. Serum total cholesterol (TC), blood glucose, triglyceride (TG), low density lipoprotein cholesterol (LDL-C) and high density lipoprotein cholesterol (HDL-C) were examined by the automatic biochemistry analyser (Hitachi 917, Tokyo, Japan).

### Hematoxylin–eosin (HE) staining

Mouse liver specimens were processed according to a standard HE staining technique [24]. Briefly, liver tissues were fixed by 10% neutral formalin, dehydrated in ethanol, and then embedded. Subsequently, liver sections (4 μm) were stained with HE for pathological changes under an optical microscope.

### Oil red O staining

Mouse liver tissues were immediately snap-frozen in liquid nitrogen and placed in OCT cryostat embedding compound (Tissue-Tek, Torrance, CA, USA). Frozen liver sections (8 μm) were stained with oil red O according to previous report [25], and the intracellular lipid droplets were observed and assessed by bright-field microscopy (Leica, Wetzlar, Germany).

### Reverse cholesterol transport study in vivo

Raw264.7 (leukemia cells in mouse macrophage) cells were obtained from American Type Culture Collection (ATCC; Manassas, Va) and were grown in DMEM supplemented with 10% fetal bovine serum. RCT was assessed in vivo by intraperitoneal injection of RAW264.7 cells that were radiolabelled with ^3^H-cholesterol according to the manufacturer’s instructions as previously described [26]. Briefly, Raw264.7 cells were radiolabeled with 5 Ci/mL ^3^H-cholesterol and cholesterol enriched with 100 g/mL of acetylated LDL for 48 h. These foam cells were washed twice, equilibrated in medium with 0.2% bovine serum albumin for 6 h, spun down, and resuspended in 0.5 mL medium. To assess the role of liraglutide in promoting efflux of cholesterol from macrophages to plasma and feces, cholesterol-loaded and ^3^H-cholesterol-labeled raw264.7 cells were injected intraperitoneally into mice and the appearance of ^3^H-cholesterol in plasma and feces over 24 h was quantified for liquid scintillation counting.

### Cell viability assay

Cell Counting Kit-8 (Dojindo Molecular Technologies, Inc. Kumamoto, Japan) was used to assess cell viability. This assay was assessed by cultivating HepG2 cells in 96-well plates for 24 h. The cells were then exposed to various concentrations of glucose (0, 5, 25, 50 and 75 mmol/L) and liraglutide (0, 10, 100, 1000 and 2000 nmol/L) for 24 h. After replacing the DMEM medium, 10 μL of CCK-8 reagent was added to each well, and the 96-well plate was incubated in the dark at 37 °C for 2 h. The absorbance was measured at 450 nm in a microplate reader. All the experiments were repeated three times.

### BODIPY-cholesterol efflux assay

BODIPY-cholesterol efflux assay was determined as previously described [27]. Briefly, a stock solution of BODIPY cholesterol was prepared at 5 mM in DMSO. HepG2 cells were loaded with 2.5 mM of BODIPY-cholesterol in culture medium for 2 h at 37 °C. Cells were rinsed twice with physiological buffer (140 mM NaCl, 5 mM KCl, 1 mM CaCl2, 1 mM MgSO4, 5 mM glucose, 20 mM HEPES, pH 7.4), and incubated with the same buffer for 1 h at 37 °C with shaking at 50 rpm. The cell supernatant was centrifuged for 5 min at 6800*g*, and the BODIPY fluorescence intensity in the supernatants was measured using a TECAN Genios Pro Microplate Reader (Tecan US, Inc., Morrisville, NC, USA) at excitation 490 ± 10 nm and emission 535 ± 20 nm.

### Real time quantitative PCR (qRT-PCR) assay

SYBR green quantitative real-time polymerase chain reaction (qRT-PCR) was used to detect mRNA levels of ABCA1, ABCG1, SR-B1. The Trizol reagent (Invitrogen, Waltham, USA) was used to extract total RNA from mouse liver tissue and HepG2 cells according to the manufacturer’s instructions and the quality of extracted RNA was measured by 260 to 280 nm absorbance. Then cDNA was obtained by reverse transcription (RT) using HifAir™ II 1st Strand cDNA Synthesis SuperMix for qPCR (YEASEN, Shanghai China). Amplification of the specific genes was performed using Hieff™ qPCR SYBR^®^ Green Master Mix (YEASEN, Shanghai China), and then the relative expression levels were analyzed with an ABI7500 real-time PCR system (Applied Biosystems). Glyceraldehyde 3-phosphate dehydrogenase (GAPDH), an endogenous housekeeping gene, was used for expression normalization. The specific RT primers and PCR primers are as follows: ABCA1 (forward 5′-ACCCACCCTATGAACAACATGA-3′ and reverse 5′-GAGTCGGGTAACGGAAACAGG-3′), ABCG1 (forward 5′-GGGGTCGCTCCATCATTTG-3′ and reverse 5′-TTCCCCGGTACACACATTGTC-3′), SR-B1 (forward 5′-CCTATCCCCTTCTATCTCTCCG-3′ and reverse 5′-GGATGTTGGGCATGACGATGT-3′), and glyceraldehyde 3-phosphate dehydrogenase (GAPDH, forward 5′-GGAGCGAGATCCCTCCAAAAT-3′ and reverse 5′-GGCTGTTGTCATACTTCTCATGG-3′). Each reaction was carried out in triplicate, and the qRT-PCR results were calculated using the previous method [28].

### Western blotting

Mouse liver tissue and HepG2 cells samples were homogenized on ice in lysis buffer [20 mM Tris–HCl, pH 7.5, 150 mM NaCl, 1 mM EDTA, 1 mM EGTA, 1% Triton X-100, 50 mM dithiothreitol, complete protease inhibitor cocktail (Roche Diag-nostics) and phosphatase inhibitor cocktail I and II (Sigma–Aldrich)]. The homogenate was then centrifuged at 12,000*g* for 15 min and the supernatant was collected. Protein concentrations were determined using a BCA Protein Assay Kit (Beijing Kangwei Century Biotechnology Co, Ltd, Beijing, China). Subsequently, 35 μg of protein from individual samples was resolved by precast NuPAGE Novex 4–12% (w/v) Bis–Tris gels (Life technologies, Carls-bad, CA, USA), and then transferred onto nitrocellu-lose membrane using the iBlotTM dry blotting system as described by the manufacturer (Invitrogen, Carlsbad, CA, USA). The membranes were blocked in TBST buffer (20 mM Tris, pH 7.5, 150 mM NaCl, 0.1% tween-20) containing 5% non-fat milk for 2 h at room temperature and then incubated overnight at 4 °C Anti-ABCA1, Anti-ABCG1 or Anti-SR-B1. Afterwards, the membranes were incubated with the secondary antibodies including goat anti-rabbit IgG/horseradish peroxidase (HRP) and goat anti-mouse IgG/HRP (Abcam) for 2 h at room temperature. Protein expression was detected with chemilumi-nescence (ECL, ermo Fisher Scienti c, Waltham, MA, USA) on FluorChem M image system.

### Statistical analysis

SPSS 19.0 (SPSS Inc., Chicago, IL, USA) and GraphPad Prism 7.0 (GraphPad software, Inc., La Jolla, CA, USA) were utilized for statistical analysis and the construction of graphs. Data was presented as mean ± standard error of the mean (SEM) unless otherwise stated. Comparisons between two groups were assessed using an unpaired two-tailed Student’s *t* test and one-way ANOVA was used for comparison of more than 2 groups, with p < 0.05 considered to be statistically significant. Unless indicated in the figure legends, all results were confirmed by at least three separate experiments.

## Results

### Liraglutide decreased blood glucose and body weight in db/db mice with high-fat diet

**Fig. 1:**
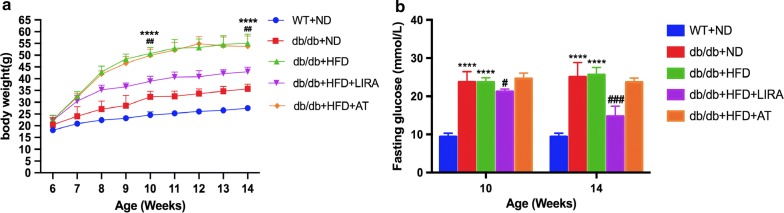
Changes in body weight (**a**) and fasting glucose (**b**) in WT mice and db/db mice. p Values are mean ± SD ****p < 0.0001: db/db + HFD vs db/db + ND or WT + ND. ^#^p < 0.05, ^##^p < 0.01, ^###^p < 0.001: db/db + HFD + LIRA vs db/db + HFD

Male db/db mice of 6 weeks fed with HFD were administered liraglutide (200 μg/kg, twice daily) for 8 weeks. As shown in Fig. 1a, b, the db/db mice fed with HFD had higher levels of body weight and fasting blood glucose at both 4 weeks and 8 weeks compared to the db/db mice fed with ND or WT mice. However, there was no significant change in fasting blood glucose levels between the db/db mice fed with HFD and those of fed with ND. As expected, liraglutide treatment significantly decreased body weight and fasting glucose levels in db/db mice fed with HFD.

### Liraglutide improved lipid metabolism in the serum and reduced lipid accumulation in the liver

**Fig. 2:**
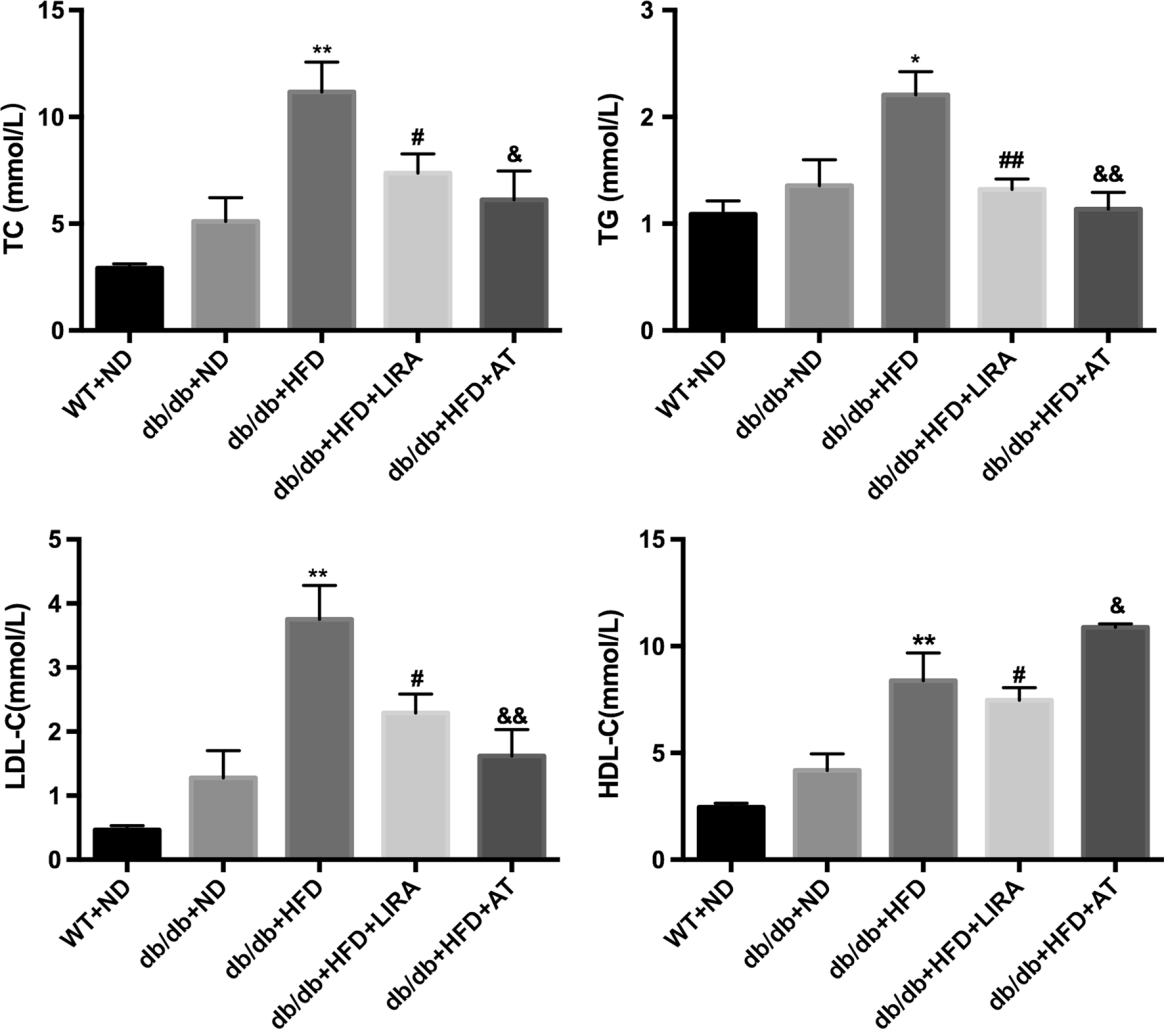
Effects of liraglutide on serum lipid profile (TC, TG, LDL-C and HDL-C) in WT mice and db/db mice. *p < 0.05, **p < 0.01: db/db + HFD vs db/db + ND. ^#^p < 0.05, ^##^p < 0.01: db/db + HFD + LIRA vs db/db + HFD. ^&^p < 0.05, ^&&^p < 0.01: db/db + HFD + AT vs db/db + HFD

After liraglutide treatment for 8 weeks, serum samples and liver tissues were respectively collected for analyzing lipid parameters and lipid accumulation. As shown in Fig. 2, the levels of TC, TG and LDL-C in the db/db mice fed with HFD were significantly higher compared to db/db mice fed with ND or WT mice. Liraglutide significantly decreased the levels of TC, TG and LDL-C similar to atorvastatin treatment. Interestingly, the data showed that liraglutide also reduced serum HDL-C concentration in db/db mice with HFD, while atorvastatin increased HDL-C level.

**Fig. 3:**
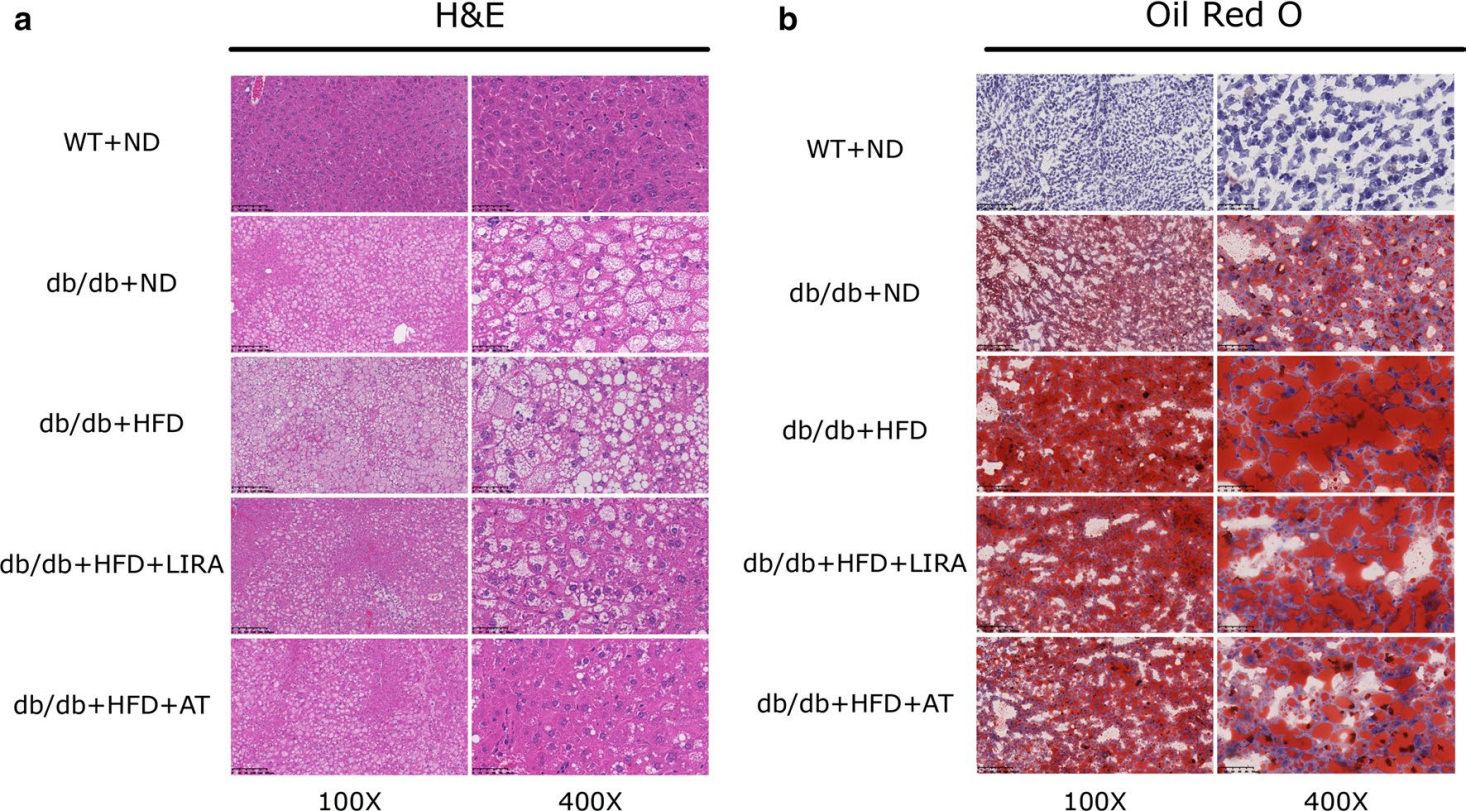
Effects of liraglutide on hepatic steatosis by staining with H&E (**a**) or Oil Red O (**b**) in WT mice and db/db mice

Furthermore, the results by HE and oil red O staining indicated that the accumulation of lipid droplets was more intensive in liver section of db/db mice fed with HFD than that of WT mice (Fig. 3). Liraglutide treatment for 8 weeks in db/db mice fed with HFD significantly reduced in the number of the lipid droplets and lipid accumulation as well as atorvastatin treatment (Fig. 3).

### Liraglutide promoted RCT in db/db mice with HFD

**Fig. 4:**
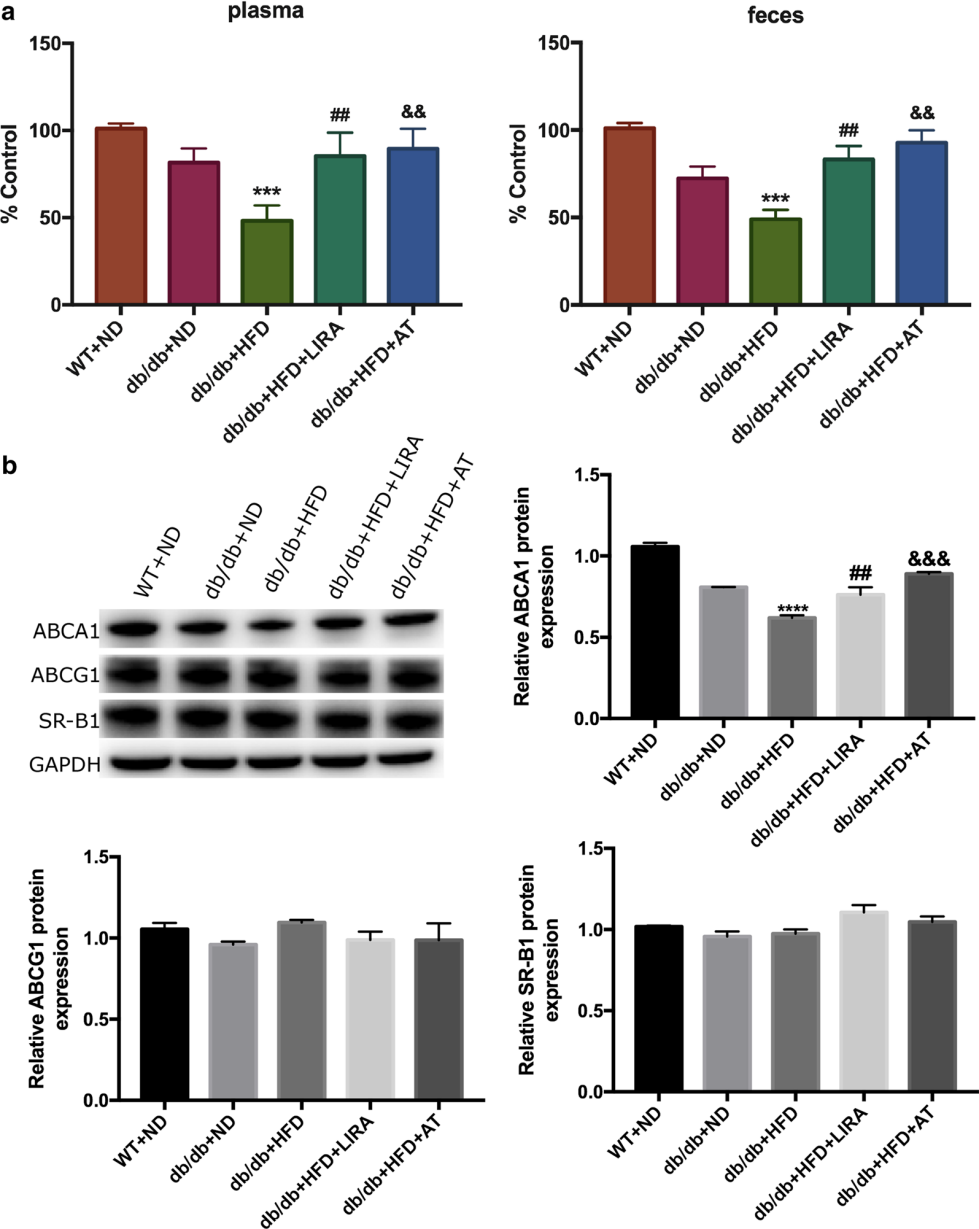
Effects of liraglutide on RCT and RCT-related protein expression in mice study. Liraglutide treatment increased the efflux of ^3^H-cholesterol from macrophages to plasma and feces in db/db mice with HFD (**a**), enhanced hepatic ABCA1 expression, and unchanged ABCG1 and SR-B1 expressions in mice (**b**). ****p < 0.0001: db/db + HFD vs db/db + ND or WT + ND ^##^p < 0.01: db/db + HFD + LIRA vs db/db + HFD. ^&&&^p < 0.001: db/db + HFD + AT vs db/db + HFD

To evaluate the role of liraglutide in regulating the efflux of cholesterol from macrophages to plasma and feces, RCT was monitored in vivo. There were significant differences in the radioactivity including serums and feces between db/db mice fed with HFD and those fed with ND, suggesting that HFD could decreased the efflux of cholesterol (Fig. 4a). Compared with db/db mice fed with HFD, a significant increase was found regarding ^3^H-cholesterol levels of the plasma and feces in those mice after liraglutide 8-week treatment (Fig. 4a). Moreover, our data also showed atorvastatin could increase the efflux of cholesterol in vivo, which was similar to previous study [29].

Furthermore, we investigated the effects of liraglutide on the proteins which were related to the process of RCT. The study suggested that db/db mice fed with HFD displayed lower expression of ABCA1 compared to those fed with ND or the WT mice (Fig. 4b). However, liraglutide treatment could significantly increase ABCA1 protein expression but no change was observed in the levels of ABCG1 and SR-B1 expression (Fig. 4b).

### Liraglutide increased cholesterol efflux in HepG2 cells under high glucose condition

**Fig. 5:**
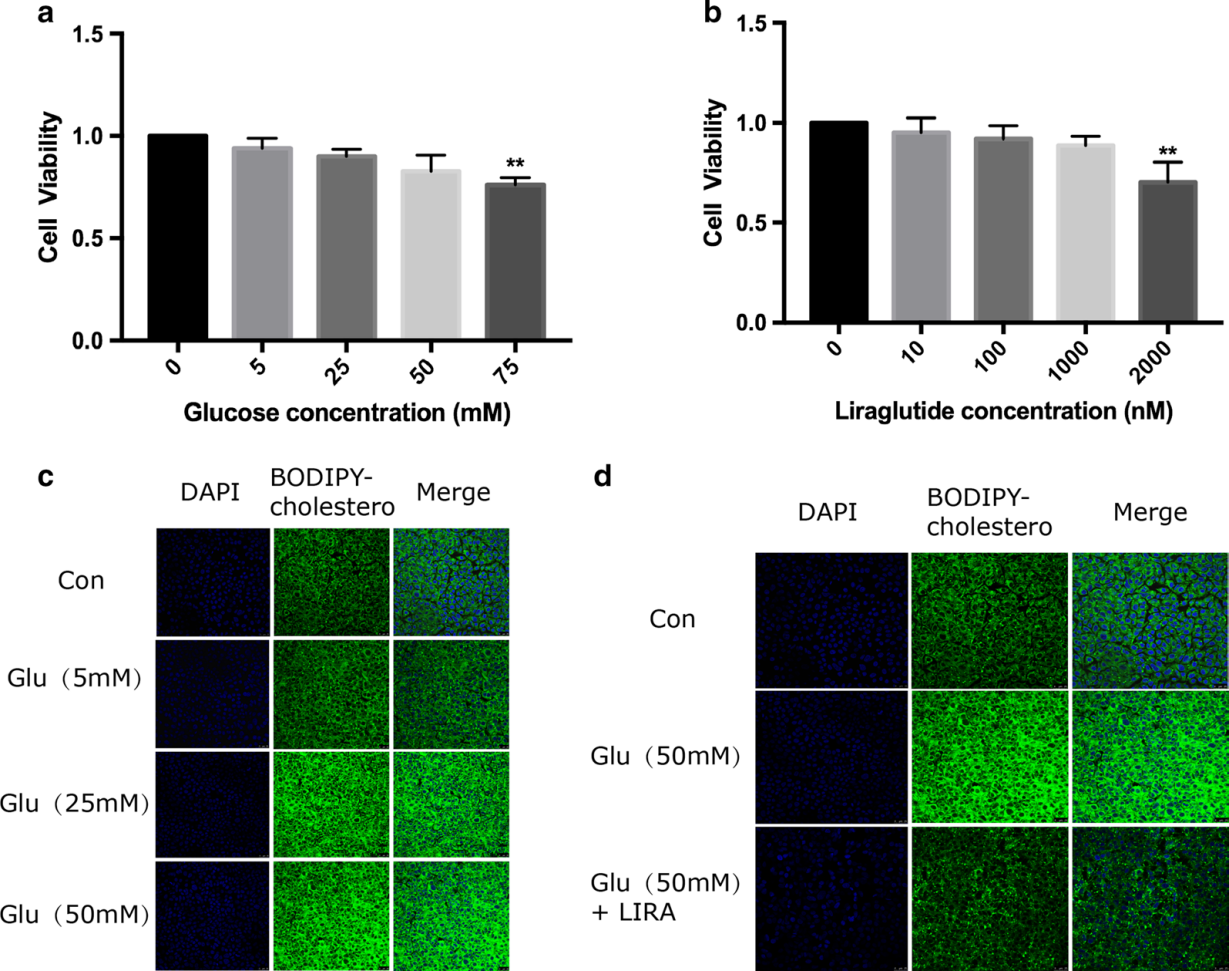
Effects of different glucose (**a**) and liraglutide (**b**) concentrations on cell viability in HepG2 cells. *p < 0.05, **p < 0.01 vs control group. Effects of different glucose concentrations on cholesterol efflux in HepG2 cells (**c**). Effect of Liraglutide (1000 nM) on cholesterol efflux in HepG2 cells under high glucose condition (**d**)

To investigate the mechanism of the effect of liraglutide on RCT in vivo, we carried out cell experiments. Firstly, we observed the effects of different concentrations of glucose and liraglutide on the activity of HepG2 cells respectively. Data showed that high glucose could reduce the viability of HepG2 cells in a dose-dependent manner (Fig. 5a). The results also showed an increased intracellular fluorescence density in a dose-dependent manner (Fig. 5c), indicating that the high glucose could decrease cholesterol efflux in HepG2 cells.

Furthermore, the impact of liraglutide on the cell viability was examined. In the primary study, we found that a 1000 nmmol/L of liraglutide had no effects on the cell viability and then used it for the next study (Fig. 5b). In order to determine the protective effect of liraglutide on cholesterol efflux in HepG2 cells under high glucose condition (50 mmol/L), we therefore selected liraglutide at a dose of 1000 nmol/L in the following experiments. Data showed that liraglutide could significantly increase intracellular cholesterol efflux in HepG2 cells under high glucose condition (Fig. 5d).

### Liraglutide up-regulated the expression of ABCA1 in HepG2 cells under high glucose condition

**Fig. 6:**
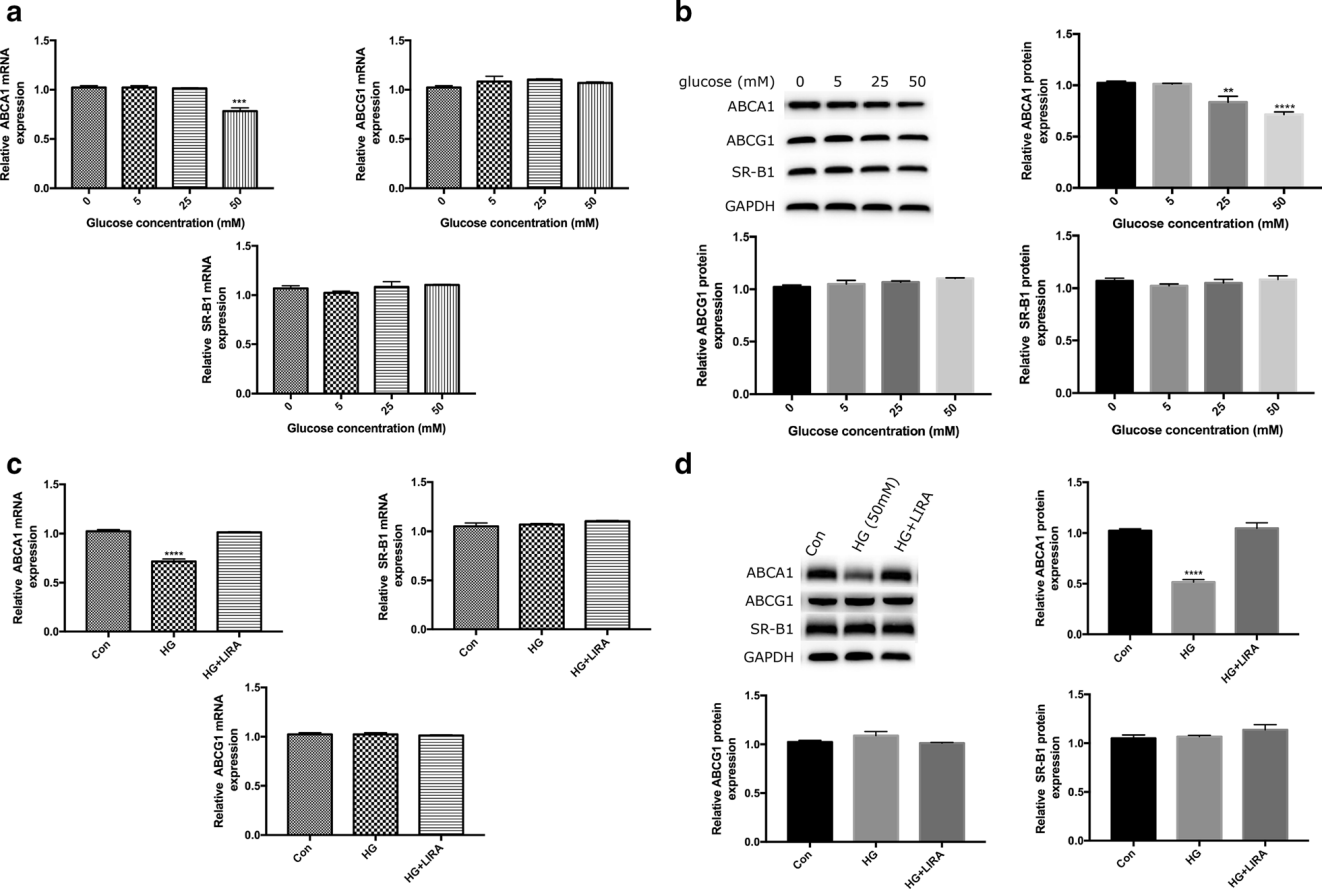
Effects of glucose at different concentrations (0, 5, 25, 50 mM) on ABCA1, ABCG1 and SR-B1 gene (**a**) and protein (**b**) expressions in HepG2 cells. Effects of liraglutide (1000 nM) on ABCA1, ABCG1 and SR-B1 gene (**c**) and protein (**d**) expressions in HepG2 cells under high glucose (HG, 50 mM) condition. **P < 0.01, ***p < 0.001, ****p < 0.0001 vs control group

To further investigate the molecular mechanism associating with the effect of liraglutide on cholesterol efflux, we treated HepG2 cells with different concentrations of glucose (0, 5, 25, 50 mmol/L) for 24 h. Our results showed that high glucose at concentration of 50 mmol/L could significantly decrease the gene expressions of ABCA1 (Fig. 6a), which pattern was also found at the protein level in a concentration-dependent manner (Fig. 6b). Furthermore, we observed liraglutide could increase both gene and protein expression of ABCA1 in HepG2 cells exposed to high glucose (Fig. 6c, d).

### Liraglutide increased ABCA1 expression by activating the ERK1/2 pathway

**Fig. 7:**
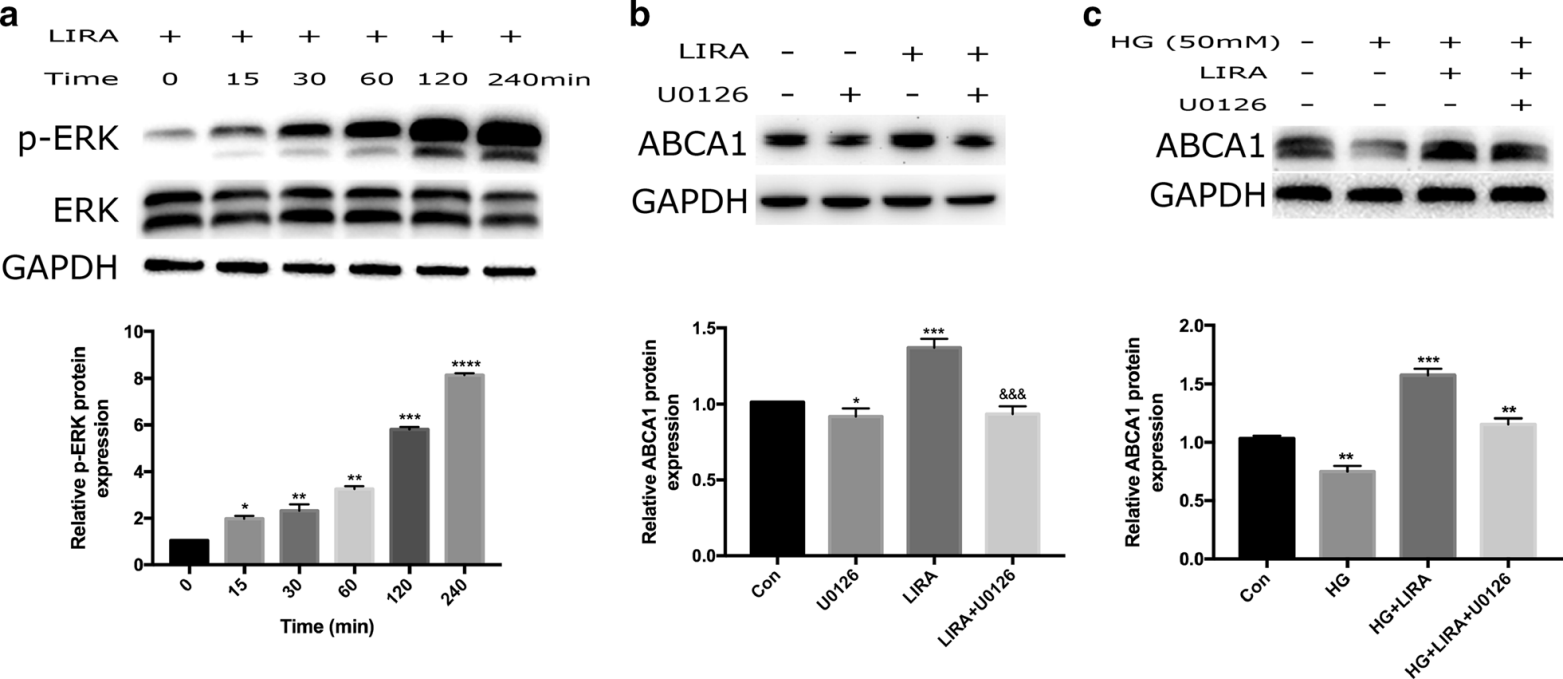
Effects of liraglutide on ERK1/2 pathway in HepG2 cells with or without high glucose. Liraglutide increased p-ERK1/2 protein expression in HepG2 cells in a time-dependent manner (**a**). U0126, the ERK1/2 inhibitor, attenuated the increased ABCA1 expression induced by liraglutide in HepG2 cells (**b**). Under high glucose (HG, 50 mM) condition, U0126 reduced the increased ABCA1 expression induced by liraglutide in HepG2 cells (**c**). *p < 0.05, **p < 0.01,***p < 0.001, ****p < 0.0001 vs control group

We next examined the possible involvement of MAPK/ERK1/2 pathway in liraglutide-induced ABCA1 expression. ERK1/2 is one member of MAPKKs family, which is an important kinase involved in many kinds of biological physiological process [30, 31]. Therefore, we investigated whether liraglutide could stimulate intracellular ERK1/2 phosphorylation events. After serum-starvation for 12 h, HepG2 cells were stimulated with liraglutide (1000 nmol/L) and cells were harvested at 0, 15, 30, 60, 120, and 240 min respectively. Activation of ERK1/2 was analyzed by Western blotting using anti-phospho-ERK1/2 antibody. Data showed that exposure of HepG2 cells to liraglutide enhanced the level of ERK1/2 phosphorylation in a time-dependent manner, which started at 15 min and peaked at 240 min (Fig. 7a). Subsequently, the results suggested that the increase in ABCA1 expression caused by liraglutide was abolished by the ERK1/2 inhibitor U0126 (50 µM), indicating that a ERK1/2 pathway might be involved in such effect (Fig. 7b). Briefly, liraglutide increased ABCA1 expression in HepG2 cells through the MAPK/ERK1/2 signaling pathway.

To further elucidate a role of ERK1/2 pathway in up-regulated ABCA1 expression by liraglutide under the condition of high glucose in HepG2 cells, the activation status of ERK1/2 was also determined. Methologically, HepG2 cells cultured under high glucose condition were pretreated for 1 h with an ERK1/2 inhibitor U0126 before the addition of liraglutide, and then harvested for Western blotting using anti-phospho-ERK1/2 antibody and anti-ERK1/2 antibody. The results indicated that liraglutide significantly increased the level of ABCA1 protein under high glucose condition in HepG2 cells, while ERK1/2 inhibitor U0126 clould reverse the effect of liraglutide on ABCA1 expression (Fig. 7c). Therefore, such data suggested that liraglutide increased ABCA1 protein expression under high glucose condition in HepG2 cells by activating ERK1/2 signaling pathway.

## Discussion

In this study, we investigated the effect of liraglutide on RCT in db/db mice and HepG2 cells. Interestingly, our data indicated that liraglutide could modify the lipid profile and promote RCT in db/db mice fed with a high-fat diet. Moreover, results also showed that liraglutide significantly reduced lipid deposition in the liver in vivo. Moreover, the present study showed that liraglutide cloud up-regulate ABCA1 expression, a key RCT-related protein, in which was associated with the activation of MAPK/ERK1/2 signaling pathway.

In fact, DM is characterized by both glucose and lipid disorders. Evidence also support the notion that DM has higher rate of ASCVD and worse clinical outcomes due to the interaction of high glucose and dyslipidemia. In this study, the hyperglycemia and hyperlipidemia models were successfully established by addition of a high-fat diet in db/db mice, which showed a persistent elevation in the levels of blood glucose and body weight. Meanwhile, serum lipid levels including TC, LDL-C and TG in diabetic mice fed with HFD were significantly higher than those fed with ND in diabetic mice and WT mice. Interestingly, liraglutide also reduced the serum HDL-C level in db/db mice fed with HFD, which is similar to my previous data [32]. More importantly, a marked inhibition of cholesterol efflux and the increased accumulation of lipid were also stably found, indicating that our model was suitable for further study regarding the impact of liraglutide on these pathophysiologic changes in the diabetic model.

As we know, liraglutide, a novel anti-diabetic medication, has recently become the first-line treatment for DM [33–35]. Previous studies have suggested that liraglutide exerts hypoglycemic effects by increasing insulin secretion, improving islet cell function, decreasing food intake, and reducing body weight [36]. In addition to down- regulation of the blood glucose, it also has the beneficial effects on the cardiovascular system [37]. A recent study showed that liraglutide had a cardiovascular protective effect in the type 2 diabetic patients presenting as a significant reduction of the cardiovascular events during a long-term follow-up [38]. Although the exact mechanism of this protective impact on cardiovascular system by liraglutide is currently not well determined, several animal and human observations have found that it may be associated with its reduction of body weight, recovery of liver lipid deposition, and reversal of hepatic steatosis [39–41]. Our previous study has demonstrated that liraglutide improves lipid metabolism by inhibiting the expression of PCSK9 in db/db mice through HNF1α-dependent mechanism and HepG2 cells [32]. Furthermore, the present study showed that liraglutide reduced the levels of TC, TG, and LDL-C in diabetic mice fed with HFD mediated by enhancing RCT.

It has been reported that RCT is a key process involving in the lipid metabolism and cardiovascular system protection. Hence, we hypothesized that the cardiovascular protective effects by liraglutide may be linked with RCT. That is the reason why we perform such study. Previous study has shown that GLP-1 may affect cholesterol homeostasis by regulating the expression of miR-758 and ABCA1 in HepG2 cells [42]. In addition, GLP-1 treatment significantly increased the expression of ABCA1, ABCG1 and LXR-α, and improved cholesterol efflux from 3T3-L1 adipocytes [43]. In our study, we found that liraglutide significantly enhanced RCT in db/db mice with high-fat diet assessed by the movement of ^3^H-cholesterol from macrophages to bloods and feces. In addition, several studies have indicated that liraglutide can induce body weight loss through reducing food intake, promoting satiety, and inducing autophagy [44–47]. In agreement with these prior studies, our data showed that liraglutide lowered blood glucose levels and body weight in db/db mice with high-fat diet.

At the same time, we also observed that liraglutide could modify TC and TG in diabetic mice, and improve hepatic lipid accumulation. Data indicated that the hepatoprotective effects of liraglutide appeared from its direct impact rather than its glucose lowering ability. Several recent studies have also showed that liraglutide can alleviate non-diabetes steatohepatitis. Ipsen et al. found that liraglutide significantly decreased hepatic inflammation, liver injury and hepatocyte ballooning in advanced lean non-alcoholic steatohepatitis in guinea pigs induced by high-fat diet [48]. The study by Zhang et al. demonstrated that liraglutide had a protective effect on carbon tetrachloride (CCl_4_)-induced acute liver injury in mice, which significantly ameliorated the liver histopathological changes, reduced hepatocyte apoptosis, and enhanced mitochondrial respiratory functions [49]. Similarly, Milani et al. found that the hepatoprotective and therapeutic effects of liraglutide on acute liver injury in mice induced by CCl_4_ might be attributable to a decrease in liver oxidative stress and the preservation of metabolism [50]. Therefore, it might be concluded that the potential hepatoprotective effect of liraglutide was beyond its glucose-lowering action.

Previous study has reported that high glucose can inhibit the expression of ABCA1 in macrophages via the ERK1/2 pathway, thereby reducing intracellular cholesterol efflux [51]. Gorgani-Firuzjaee et al. showed that high glucose can induce de novo synthesis of cholesterol and VLDL production in HepG2 cells [52]. Pang et al. have found that long-term exposure of HepG2 cells to high glucose can induce reactive oxygen species (ROS) accumulation and DNA damage [53]. Do et al. have reported that high glucose can induce lipid accumulation in HepG2 cells [54]. Similarly, our study demonstrated that high glucose (50 mmol/L) could significantly reduce ABCA1 expression and inhibit HepG2 cells cholesterol efflux. It should be mentioned that signal transduction pathway regarding the role of liraglutide in regulating RCT is currently unclear although we found a beneficial impact of liraglutide on RCT in animal. As described in the section of introduction, there are at least three key mediators (ABCA1, ABCG1 and SR-B1) involving in RCT process. In order to explore which mediator is mainly associated with the RCT by liraglutide, we established a high glucose-stimulated HepG2 cell model. By using this model, we confirmed the exact role of liraglutide in enhancing cell cholesterol efflux. We subsequently examined the effects of liraglutide on the expression of the mediators ABCA1, ABCG1 and SR-B1 and related signaling pathways. Data suggested that liraglutide up-regulated ABCA1 expression mediated by ERK1/2 phosphorylation, resulting in cholesterol efflux increase in HepG2 cells under the high glucose conditions. Our findings may be an important complementary information concerning the relation of liraglutide to lipid metabolism.

## Conclusions

In conclusion, the present study firstly indicated that liraglutide could improve lipid metabolism and decrease hepatic lipid accumulation in db/db mice fed with high-fat diet through promoting the process of RCT, which was associated with ABCA1 mediated by activating MAPK/ERK1/2 signal pathway. These results may widen our knowledge regarding the role of liraglutide in cardiovascular medicine beyond its glucose-lowering action.

## Data Availability

All data generated or analyzed during this study are included in this published article.

## Abbreviations

RCT: reverse cholesterol transport
HepG2: human hepatoma cell
Raw264.7: leukemia cellsin mouse macrophage cell
HFD: high fat diet
TC: total cholesterol
TG: triglyceride
LDL-C: low-density lipoprotein cholesterol
HDL-C: high density lipoprotein cholesterol
DM: diabetes mellitus
AS: atherosclerosis
ASCVD: atherosclerotic cardiovascular disease
ABCA1: ATP binding cassette transporter A1
ABCG1: ATP binding cassette transporter G1
SR-BI: scavenger receptor B1
HDL: high-density lipoprotein
GLP‐1: glucagon‐like peptide‐1
LIRA: liraglutide
AT: atorvastatin
ND: normal diet
WT: wild type
